# Characterization of Gut Microbiome Composition in Depression and Completed Suicide

**DOI:** 10.3390/ijms26104880

**Published:** 2025-05-19

**Authors:** Samat Kozhakhmetov, Alibek Kossumov, Tolkyn Zhakupova, Tatyana Polyakova, Nazgul Imambayeva, Bagyzhan Syzdykova, Aidana Rakhmankulova, Gulshat Dalibayeva, Artur Kovenskiy, Zharkyn Jarmukhanov, Argul Issilbayeva, Elizaveta Vinogradova, Almagul Kushugulova

**Affiliations:** 1 Laboratory of Microbiome, Center for Life Sciences, National Laboratory Astana, Nazarbayev University, 53 Kabanbay Batyr Ave., Block S1, Astana Z05H0P9, Kazakhstan; alibek.kossumov@nu.edu.kz (A.K.); aidana.rakhmankulova@nu.edu.kz (A.R.); artur.kovenskiy@nu.edu.kz (A.K.); zharkyn.jarmukhanov@nu.edu.kz (Z.J.); argul.issilbayeva@nu.edu.kz (A.I.); st.paulmississippi@gmail.com (E.V.); akushugulova@nu.edu.kz (A.K.); 2 Kazakhstan Society of Human Microbiome Researchers, Astana Z00T2C6, Kazakhstan; 3 Department of Forensic Medicine, NJSC, Astana Medical University, Astana Z01G6C5, Kazakhstan; tolkin1508@gmail.com (T.Z.); pti80@list.ru (T.P.); syzdykova-75@mail.ru (B.S.); 4 Research Institute of Forensic Examinations of the State Enterprise, Center for Forensic Examinations of the Ministry of Justice of the Republic of Kazakhstan, Astana Z00X8P9, Kazakhstan; imambaevan@mail.ru; 5 Multidisciplinary City Hospital No. 1, Astana Z01F7K6, Kazakhstan; 6 Interdisciplinary Sports Research, Center for Genetics and Life Sciences, Sirius University of Science and Technology, 1 Olympic Ave., Sirius Federal Territory, Sochi 354340, Russia

**Keywords:** gut microbiome, depression, suicide, gut–brain axis, *Firmicutes*, *Lachnospiraceae*, metabolic pathways, energy metabolism, inflammation, mental health

## Abstract

Growing evidence supports a bidirectional relationship between the gut microbiome and mental health. This study investigated the association between gut microbiota, depression, and suicidal behavior by analyzing fecal samples from 35 individuals with varying depression levels and 36 completed suicide cases. Standardized psychometric assessments were used for depression evaluation. Analysis revealed significant taxonomic differences between groups, with increased abundance of *Firmicutes*, *Clostridia*, *Lachnospiraceae*, *Blautia*, and *Dorea* in suicide cases, which also positively correlated with depression severity. Metabolic pathway analysis demonstrated a notable dichotomy: suicide cases showed elevated pathways related to infection processes, inflammation, and antibiotic resistance, while the control group exhibited higher energy metabolism and vitamin synthesis pathways. The findings establish specific microbiome profiles associated with both depression symptoms and suicidal behavior, suggesting that gut dysbiosis may influence mental health through altered energy metabolism and inflammatory processes, potentially offering new perspectives for diagnostic and therapeutic approaches.

## 1. Introduction

A growing body of scientific evidence supports the existence of bidirectional communication between the gut microbiome and the central nervous system, termed the “gut-brain axis”. This complex system of interactions exerts substantial influence on emotional states, behavior, and mental health. Of particular interest are studies demonstrating a significant association between gut dysbiosis and the development of depressive disorders.

Numerous investigations have identified specific alterations in gut microbiota composition among patients with major depressive disorder (MDD) compared to healthy individuals. These changes are characterized by disruptions in the balance between major bacterial phyla, including *Firmicutes*, *Actinobacteria*, and *Bacteroidetes* [1,2]. Notably, fecal microbiota transplantation from MDD patients to germ-free mice induced depressive-like behaviors, suggesting not merely an association but a potential causal relationship between gut dysbiosis and depression [1].

At the taxonomic level, certain bacterial genera have been linked to depressive symptoms, including *Eggerthella*, *Subdoligranulum*, *Coprococcus*, and *Blautia* [3,4]. Many of these microorganisms participate in the synthesis of neurotransmitters and metabolites critical for mood regulation. Of particular interest are short-chain fatty acids produced by gut bacteria, as well as bacterial strains capable of synthesizing or modulating levels of serotonin, gamma-aminobutyric acid (GABA), and other neuroactive substances [5].

Depression represents one of the most prevalent psychiatric disorders, affecting approximately one in ten men and one in four women during their lifetime. Approximately 70% of patients diagnosed with depression face the risk of disease recurrence. Particularly concerning is the association between depression and suicide: 11–17% of suicide cases are linked to depressive disorders, while 60–70% of individuals who commit suicide suffer from significant depression [6].

Kumar et al. (2023) note that by 2030, depression may surpass heart failure to become the most prevalent disease worldwide. Anxiety and depressive disorders frequently coexist, with anxiety disorders developing in 47–58% of cases during a depressive episode, while 56% of individuals with anxiety disorders experience depression [7].

The mechanisms of interaction between gut microbiota and the brain include influences on neurogenesis, blood–brain barrier integrity, neuroinflammation, and hypothalamic–pituitary–adrenal axis functioning [8]. Dysbiosis and increased intestinal barrier permeability may lead to systemic inflammation and oxidative stress, which, in turn, contribute to the development of depression and other psychiatric disorders [9].

As noted, the gut microbiome is closely connected to brain function through the gut–brain axis. GABA, secondary bile acids, short-chain fatty acids, and tryptophan metabolites produced by the microbiota are key molecules regulating this relationship. When gut microbiome composition is disrupted, dysregulation of the gut–brain axis occurs, which is associated with neuroinflammation and altered blood–brain barrier permeability [7].

Of particular concern is the association between microbiome disturbances and suicidal behavior. Recent studies indicate that certain enterotypes and microbiome alterations may be associated not only with depressive symptoms but also with an increased risk of suicidal ideation. This relationship may be influenced by adverse childhood experiences and the recurrent nature of depressive episodes, which also impact gut microbiota composition [10].

These complex interrelationships open new perspectives for the prevention and treatment of depression through targeted microbiome modulation. Therapeutic strategies aimed at correcting the microbiome, including the application of probiotics, prebiotics, and specialized dietary interventions, demonstrate promising results in experimental and clinical studies [5,8].

In the present study, we aim to deepen the understanding of the relationship between gut microbiome, depression, and suicidal behavior by examining specific microbiome profiles in patients with depressive disorder and cases of completed suicide. We hypothesize that identifying specific microbial markers and associated metabolic pathways may contribute to the development of novel diagnostic and therapeutic approaches for these severe conditions.

## 2. Results

The study included 36 samples from cases of completed suicide (the “Deceased case” group) and 35 samples from living volunteers (the “Alive cntrl” group). The “Alive cntrl” group included both participants with varying degrees of depressive symptomatology (n = 17) and healthy participants without signs of depression (n = 18) according to the administered psychometric tests. Samples for the “Deceased case” group were obtained by forensic medical examiners from the Branch of the Institute of Forensic Examinations of the Center for Forensic Examinations of the Ministry of Justice of the Republic of Kazakhstan immediately after bodies were received. Fecal samples in the “Alive cntrl” group were collected within 2 days following psychometric assessment by clinical specialists.

**Figure 1:**
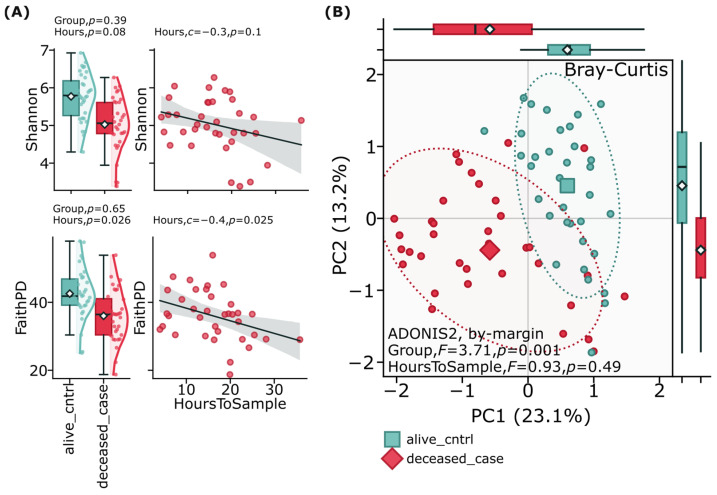
Alpha and beta diversity analysis of gut microbiome in suicide cases versus living controls. (**A**) shows alpha diversity metrics between groups, with boxplots representing Faith’s Phylogenetic Diversity (Faith’s PD) and Shannon diversity index. No significant (*p* > 0.05) differences were observed in alpha diversity metrics between the deceased case group and alive control group. (**B**) illustrates beta diversity analysis through Principal Coordinate Analysis (PCoA) based on Bray–Curtis distances, reflecting quantitative differences in community structure. The circles in the graph (**B**) represent individual samples: blue ones are from the control group of living participants, red ones are suicide cases. The dotted line shows the Bray-Curtis distance clustering boundary, visualizing differences in microbiome community structure between groups. Statistical significance was determined using PERMANOVA with 999 permutations, adjusting for age, sex, and time between death and sample collection.

Alpha and beta diversity analyses of the gut microbiome revealed important patterns between suicide cases and controls. Alpha diversity metrics, including Faith’s Phylogenetic Diversity (Faith’s PD) and the Shannon diversity index, were assessed to evaluate overall microbial diversity within each group, showing no significant (*p* > 0.05) differences between suicide cases and controls (Figure 1A). Beta diversity analysis using Bray–Curtis distance metrics was employed to examine compositional differences between groups (Figure 1B). This analysis demonstrated clear separation between the deceased case and alive control groups (F = 3.71, *p* = 0.001) when visualized through principal coordinate analysis (PCoA). These diversity patterns persisted after adjusting for confounding factors including age, sex, and time between death and sample collection, suggesting that while the overall diversity of bacterial species might be comparable between groups, the composition and relative abundance of the gut microbiome significantly differs between individuals who died by suicide and living controls.

Psychoactive substance use was most prevalent in the completed suicide group: 44.44% consumed alcohol, 8.33% used narcotic substances, and 61.11% smoked. The control group with depressive disorders exhibited lower rates: 8.57% consumed alcohol and 5.71% smoked.

In the completed suicide group, the primary method was mechanical asphyxiation (hanging)—86.11%, followed by falls from height (11.11%) and other methods (2.77%).

In our study, we employed a correlational approach to identify associations between the representation of various gut microbiome taxa and the severity of depressive and anxiety symptoms, assessed using standardized psychometric scales. As shown in Table 1, the “Alive cntrl” group demonstrated considerable diversity in the severity of depressive symptoms ranging from absence of depression to moderate and severe forms according to various scales.

**Figure 2:**
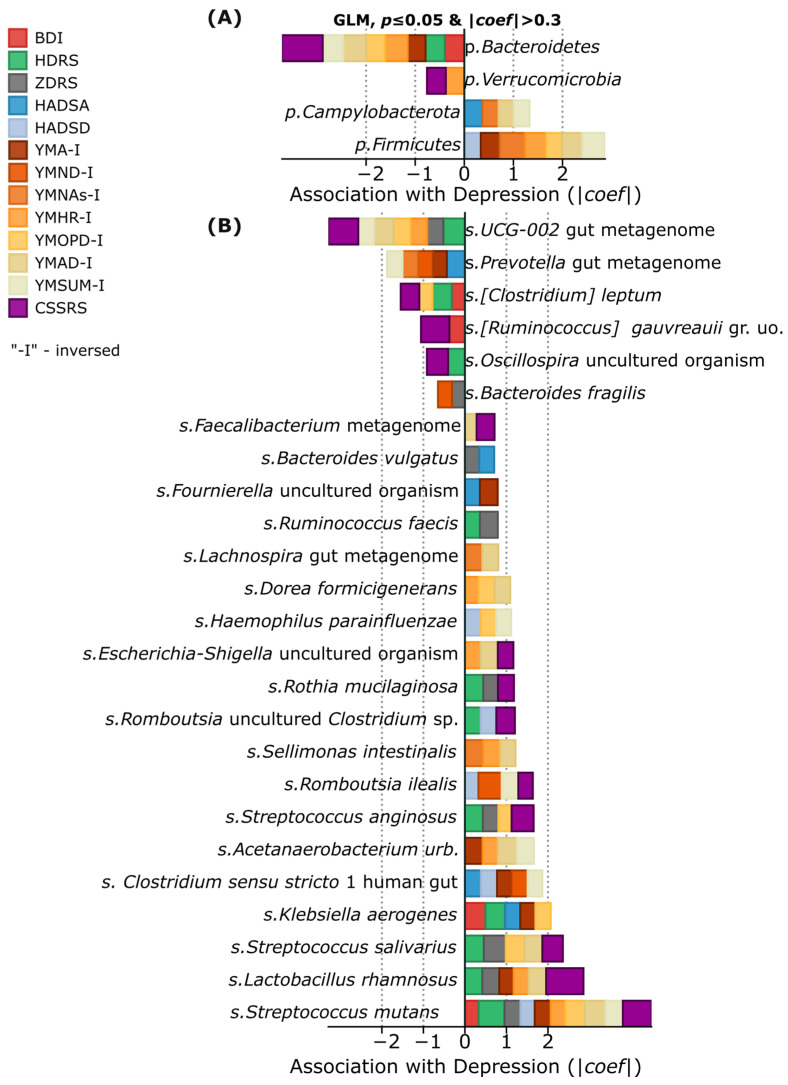
Associations between gut microbiome bacteria at different taxonomic levels and indicators of depression/suicidal intentions. (**A**) Associations between phylum-level bacterial taxa and depression assessment scales. The stacked barplot displays significant correlations (*p* ≤ 0.05) with absolute coefficient values above 0.3 for major bacterial phyla. (**B**) Associations between species-level bacterial taxa and depression metrics. Each colored bar represents a different depression and anxiety assessment scale, with the horizontal axis showing the strength and direction of relationships.

Analysis of gut microbiome composition in patients with depressive disorder revealed specific bacterial signatures associated with depression and suicidal behavior. It is important to note that the “Alive cntrl” group did not include patients with recent suicide attempts; however, suicidal intentions were identified in three participants according to the CSSRS scale. Positive correlations with the severity of depressive symptoms were found for several representatives of gram-positive bacteria, including *Streptococcus mutans*, *Streptococcus salivarius*, and *Lactobacillus rhamnosus*. At the phylum level, *Firmicutes* and *Campylobacterota* showed the most stable positive associations with depression indicators. Conversely, representatives of the phyla *Verrucomicrobia* and Bacteroidetes (particularly UCG-002, *Prevotella*, *Clostridium leptum*, *Ruminococcus gauvreauii*, *Oscillospira*, and *Bacteroides fragilis*) demonstrated negative correlations, which may indicate their potential protective effect (Figure 2A). Especially strong associations with suicidal intentions according to the CSSRS scale were identified for *Streptococcus mutans*, *Streptococcus salivarius*, and *Lactobacillus rhamnosus*, as well as *Streptococcus anginosus*, representatives of the genus *Romboutsia* (*Romboutsia ilealis* and *Romboutsia uncultured_Clostridium* sp.), *Rothia mucilaginosa*, and uncultivated representatives of *Escherichia-Shigella* (Figure 2B). This distribution of taxa suggests the existence of specific microbial patterns associated with depression and suicide risk as measured by psychometric scales.

**Figure 3:**
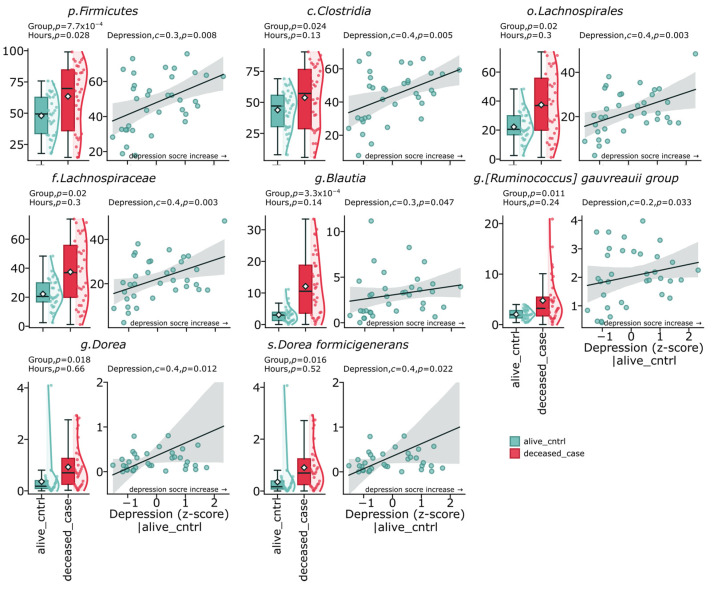
Intersection between taxonomic markers of depression and suicide. The diagrams show significant increases in specific taxa in suicide cases, including representatives of the *Firmicutes* phylum, *Clostridia* class, *Lachnospiraceae* family, and the genera *Blautia* and *Dorea*. These same taxa demonstrate positive correlations with depression indicators in the control group.

The study results demonstrate a distinct relationship between gut microbiome composition, depressive states, and suicide cases. Analysis of the fecal microbiome revealed significant differences between the deceased group (deceased_case) and the control group (alive_cntrl). The most pronounced differences were found for representatives of the phylum *Firmicutes* (*p* = 0.00077), which also demonstrated a positive correlation with depression indicators (c = 0.3, *p* = 0.008). The class *Clostridia* showed a significant positive correlation with depression levels (c = 0.4, *p* = 0.005) and was significantly elevated in the suicide group (*p* = 0.024). The order *Lachnospirales* and the family *Lachnospiraceae* were significantly elevated in the deceased group (*p* = 0.02) and positively correlated with the severity of depressive symptoms (c = 0.4, *p* = 0.003). At the genus level, the most statistically significant difference between groups was identified for *Blautia* (*p* = 0.00033), which also had a positive correlation with depression (c = 0.3, *p* = 0.047). The genus *Dorea* and its representative *Dorea formicigenerans* demonstrated a significant increase in the deceased group (*p* = 0.018 and *p* = 0.016, respectively) and a positive correlation with depressive symptoms (c = 0.41, *p* = 0.012 and c = 0.4, *p* = 0.022). The group *Ruminococcus gauvreauii* was also elevated in the deceased group (*p* = 0.011) and showed a positive correlation with depression (c = 0.2, *p* = 0.033) (Figure 3).

The presented data indicate the existence of specific microbiome profiles associated with depressive states and suicidal behavior, with the most pronounced positive associations demonstrated by representatives of the phylum *Firmicutes*, especially from the family *Lachnospiraceae*, as well as several streptococci.

**Figure 4:**
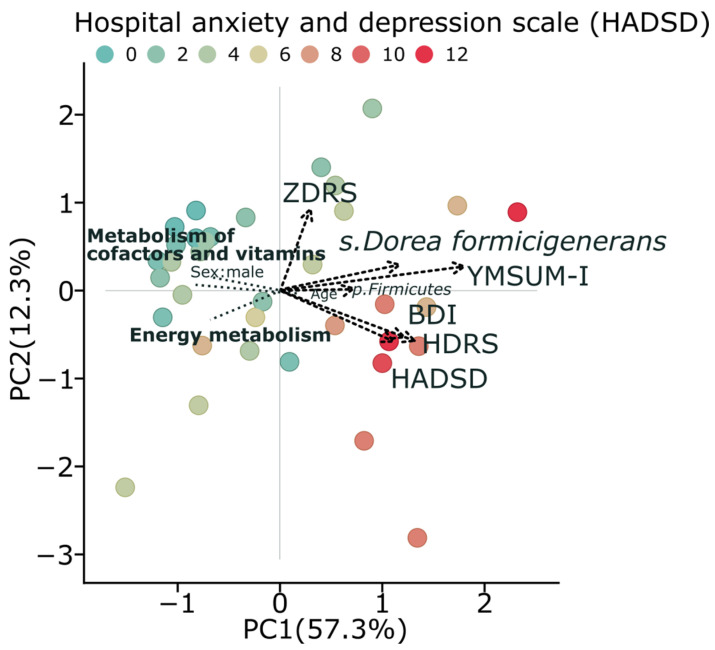
Principal Component Analysis (PCA) decomposition of psychometric scales demonstrates a high degree of concordance. The plot shows the first two principal components, accounting for 57.3% and 12.3% of variance, respectively. Points are colored according to Hospital Anxiety and Depression Scale Depression subscale (HADSD) scores, with darker red indicating higher depression severity. Depression assessment scores (HADSD, BDI, HDRS, YMSUM-inv) cluster together and positively correlate with bacterial taxa, particularly *Dorea formicigenerans* and phylum *Firmicutes*, while negatively associating with energy metabolism and metabolism of cofactors and vitamin pathways. This visualization demonstrates the inverse association between depression severity and energy-related metabolic pathways, while highlighting positive associations with specific bacterial taxa.

For further investigation, we combined all psychometric scales into one parameter using Principal Component Analysis (PCA) (Figure 4). The first PCA component explained 57.3% of the total variation and was used to represent a robust relative “depression scale”.

PCA analysis demonstrates clear patterns in the relationships between microbial communities, their metabolic functions, and depression. Subjects with higher depression scores (darker red points as colored by HADSD) are predominantly located in the positive region of PC1, which represents a higher summary score for depression and correlates with increased abundance of *Dorea formicigenerans* and *Firmicutes*. Conversely, healthier metabolic profiles, characterized by energy metabolism and metabolism of cofactors and vitamins, are positioned in the opposite direction, with the negative correlation with depression severity observed in our analyses. The clustering of multiple depression assessment tools (HADSD, BDI, HDRS) in the same area of the graph indicates the consistency of these relationships across different measurement instruments (Figure 4). This integrated analysis supports our findings regarding the dichotomy of metabolic pathways between depressive and control subjects, with a notable reduction in energy metabolism in individuals with higher depression scores.

**Figure 5:**
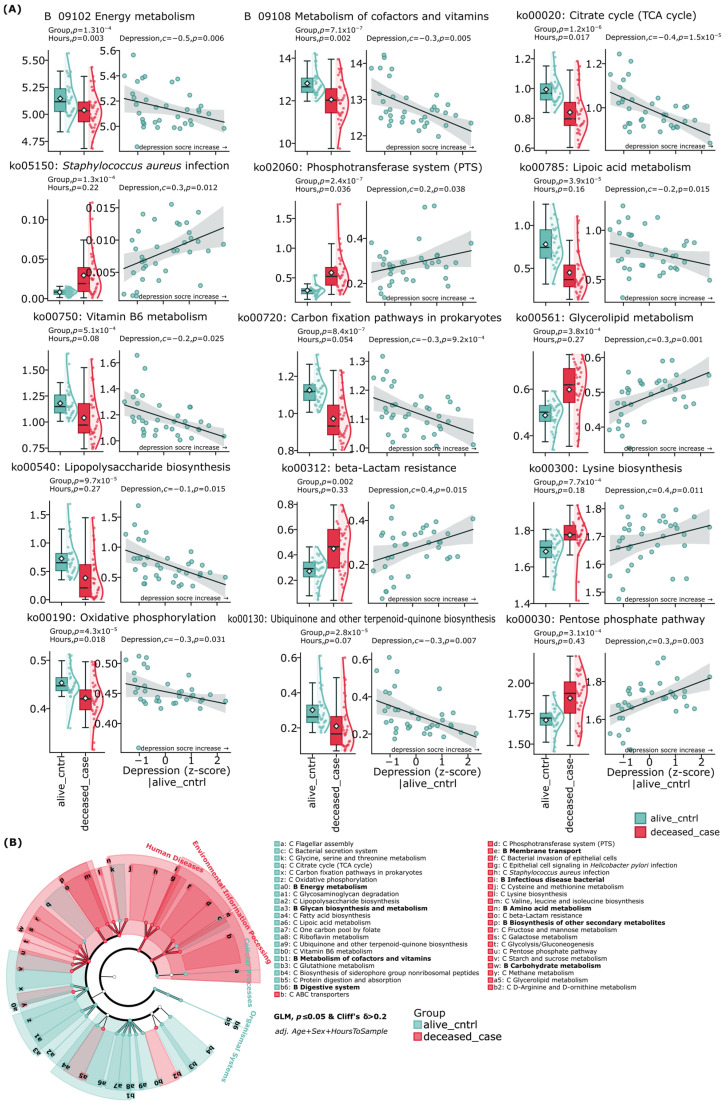
Analysis of predicted metabolic pathways comparing control subjects and completed suicide cases. (**A**) Intersection between metabolic markers of depression and suicide. (**B**) The diagrams show differences in the functional potential of the gut microbiome between groups. Red color indicates metabolic pathways significantly elevated in the suicide group, while teal blue color indicates pathways predominant in the control group.

KEGG pathway analysis revealed bidirectional alterations in the functional potential of the gut microbiome in depression and suicide cases (Figure 5). The deceased group exhibited a significant increase in the following predicted metabolic pathways, which also positively correlated with depression indicators: pentose phosphate pathway (*p* < 0.001, c = 0.3, *p* = 0.003), metabolic pathways associated with Staphylococcus aureus infection (*p* < 0.001, c = 0.3, *p* = 0.012), glycerolipid metabolism (*p* = 0.00038, c = 0.3, *p* = 0.001), phosphotransferase system (PTS) (*p* = 2.4 × 10^−7^, c = 0.2, *p* = 0.038), beta-lactam resistance (*p* = 0.002, c = 0.4, *p* = 0.015), and lysine biosynthesis (*p* < 0.001, c = 0.4, *p* = 0.011).

In contrast, the control group was characterized by increased representation of pathways negatively correlating with depression: tricarboxylic acid cycle (*p* < 0.001, c = −0.4, *p* < 0.001), lipoic acid metabolism (*p* < 0.001, c = −0.2, *p* = 0.015), lipopolysaccharide biosynthesis (*p* < 0.001, c = −0.1, *p* = 0.015), oxidative phosphorylation (*p* < 0.001, c = −0.3, *p* = 0.031), vitamin B6 metabolism (*p* < 0.001, c = −0.2, *p* = 0.025), ubiquinone and other terpenoid-quinone biosynthesis (*p* < 0.001, c = −0.3, *p* = 0.007), carbon fixation pathways in prokaryotes (*p* < 0.001, c = −0.3, *p* = 9.2 × 10^−4^), as well as general pathways of energy metabolism (*p* < 0.001, c = −0.5, *p* = 0.006) and metabolism of cofactors and vitamins (*p* < 0.001, c = −0.3, *p* = 0.005).

Among the identified correlations, the most pronounced negative associations with depression were demonstrated by the Krebs cycle (c = −0.4, *p* < 0.001) and energy metabolism (c = −0.5, *p* = 0.006), whereas the strongest positive correlations were observed for beta-lactam resistance pathways (c = 0.4, *p* = 0.015) and lysine biosynthesis (c = 0.4, *p* = 0.011).

The circular diagram of metabolic pathways clearly illustrates the identified dichotomy: the deceased group is dominated by pathways associated with infectious processes, inflammation, and antibiotic resistance, while the control group shows prevalence of energy metabolism and vitamin synthesis pathways. This pattern of functional changes may reflect potential mechanisms of the relationship between gut dysbiosis and the pathophysiology of depression and suicidal behavior.

**Figure 6:**
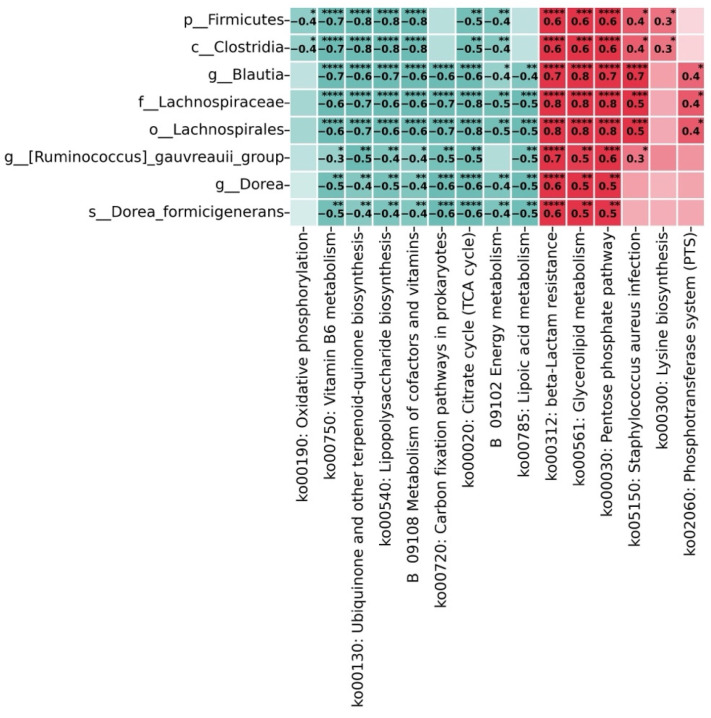
Correlation heatmap between marker bacterial taxa significantly associated with suicide/depression and their predicted metabolic pathways. Teal/blue colors indicate negative correlations, while red colors represent positive correlations, with color intensity corresponding to correlation strength. Statistical significance levels are denoted by asterisks: * *p* < 0.05, ** *p* < 0.01, *** *p* < 0.001, **** *p* < 0.0001.

Bacteria of the phylum *Firmicutes*, class *Clostridia*, family *Lachnospiraceae*, order *Lachnospirales*, as well as genera *Blautia*, *Dorea*, *Ruminococcus gauvreauii* group, and the species *Dorea formicigenerans* demonstrate strong negative correlations (teal blue color, values from −0.3 to −0.8) with metabolic pathways related to energy metabolism: oxidative phosphorylation, vitamin B6 metabolism, terpenoid-quinone biosynthesis, lipopolysaccharide biosynthesis, metabolism of cofactors and vitamins, carbon fixation pathways, Krebs cycle, energy metabolism, and lipoic acid metabolism (Figure 6). Simultaneously, these same taxa show positive correlations (red color, values from 0.3 to 0.8) with metabolic pathways associated with pathogenic processes: beta-lactam resistance, glycerolipid metabolism, pentose phosphate pathway, *Staphylococcus aureus* infection, lysine biosynthesis, and the phosphotransferase system (PTS).

The strongest negative correlations are observed between the family Lachnospiraceae and carbon fixation pathways (−0.7, *p* ≤ 0.0001) and the Krebs cycle (−0.8, *p* ≤ 0.0001), as well as between the order Lachnospirales and the Krebs cycle (−0.8, *p* ≤ 0.0001). The most pronounced positive correlations are noted between the order Lachnospirales and beta-lactam resistance (0.8, *p* ≤ 0.0001), glycerolipid metabolism (0.8, *p* ≤ 0.0001), and the pentose phosphate pathway (0.8, *p* ≤ 0.0001), as well as between the family Lachnospiraceae and these same metabolic pathways (0.8, *p* ≤ 0.0001).

The heatmap demonstrates the functional connection between changes in microbiome composition and metabolic pathways, supporting the hypothesis about potential mechanisms of interaction between gut dysbiosis, disruptions in energy metabolism, and inflammatory processes in depression and suicidal behavior.

## 3. Discussion

Our study demonstrates a clear relationship between gut microbiome composition, depression, and suicidal behavior, providing new data on potential biological markers and mechanisms underlying these conditions. We conducted a comprehensive analysis, including not only cases of clinical depression but also completed suicide, which allowed us to identify a microbiome continuum from normal to extreme pathology.

The connection between the gut microbiome and the central nervous system through the “gut-brain axis” is increasingly recognized as bidirectional and complex. As noted, there are multiple communication pathways between gut bacteria and the brain, including signaling via the vagus nerve, immune system modulation, and production of neuroactive compounds. Our results support this concept, demonstrating specific taxonomic and functional signatures associated with depression and suicidal behavior. The increased representation of the phylum *Firmicutes*, class *Clostridia*, family *Lachnospiraceae*, and genus *Blautia* in the deceased group is consistent with previous studies of depression [3,4] but demonstrates more pronounced changes in suicidal behavior. Notably, the genus *Blautia*, which showed the most statistically significant difference between groups (*p* = 0.00033), has previously been associated with depression in several studies [11,12], but its connection to suicidal behavior is demonstrated here for the first time.

Unlike the study by Kelly et al. (2016), which reported decreased microbiome diversity in depression, our results indicate specific taxonomic shifts rather than an overall decrease in diversity [13]. This may suggest that it is not so much the overall diversity, but the functional potential of certain bacterial communities that plays a key role in the pathophysiology of depression and suicidal behavior. According to their research, 60–70% of people who commit suicide have a history of significant depression [6], which is also confirmed by our observations regarding microbiome changes in both the depression group and the group with fatal suicidal outcomes.

It is noteworthy that similar alterations in gut microbiota composition are observed not only in adults but also in adolescents with depression. A recent investigation by Cheng et al. (2025) demonstrated comparable patterns of decreased *Faecalibacterium* and elevated pro-inflammatory bacteria, such as *Streptococcus*, in adolescents with major depressive disorder, corroborating the universality of these microbiome alterations irrespective of age and suggesting common gut–brain axis interaction mechanisms in depression pathogenesis across different life stages [14].

The identified association between the genus *Dorea*, its representative *Dorea formicigenerans*, and depressive/suicidal states deserves special attention. Chen et al. (2019) in a recent meta-analysis also noted an increased representation of *Dorea* in depression [15]; however, our study is the first to establish a connection between this taxon and suicidal behavior, as well as to demonstrate a significant correlation with the severity of depressive symptoms (c = 0.41, *p* = 0.012). Although some representatives of the genus *Dorea* are capable of producing gamma-aminobutyric acid (GABA) [16], the paradoxical increase in their representation in depression may be explained by compensatory mechanisms or the production of other metabolites with pro-inflammatory effects, which requires further study.

The association between Streptococcus species and depression/suicidal behavior in our study may reflect the influence of dietary factors, particularly sugar. Emerging research suggests sugar functions similarly to addictive substances like tobacco and alcohol [17], with depressed individuals often showing increased consumption patterns [18]. This may selectively promote sugar-metabolizing bacteria, including Streptococcus, explaining both their increased abundance and the enhanced pentose phosphate pathway activity we observed in suicide cases. Moreover, environmental factors like intestinal pH can influence bacterial strain functionality without changing taxonomy. For instance, GABA production by certain bacterial strains is pH-dependent [16], and sugar consumption can alter intestinal pH through fermentation. This strain-shifting phenomenon may contribute to the metabolic dichotomy we identified between groups, where pathways related to energy metabolism were reduced and inflammatory pathways enhanced in suicide cases, potentially reflecting consequences of high sugar dietary patterns.

Our findings on gut microbiome composition in depression and suicidal behavior should be considered within the context of broader human microbiota research. Studies by Wingfield et al. (2021) and Ahrens et al. (2022) demonstrated altered abundance of Veillonella in the oral microbiome of patients with depression and suicidal ideation, respectively [19,20]. The oral–brain axis research suggests that microbiota-mediated inflammatory processes can directly impact central nervous system functions [21]. Recently, Chen and Wu (2025) identified a significant association between the genus Phascolarctobacterium and suicidal ideation in major depressive disorder patients [22], further substantiating the gut microbiota’s role in suicidal behavior pathogenesis. Given our identified associations between Streptococcus and suicide risk, future investigations should examine the interrelationship between oral and gut microbiota in mental health to develop novel diagnostic and therapeutic approaches.

The microbiome signatures identified in our study may serve as potential biomarkers for disease severity and progression. Recent reviews have indicated that dysbiotic oral microbiome profiles show promise as biomarkers for various neurological and psychiatric conditions [23]. A multi-site approach combining oral and gut microbiome data could enhance diagnostic accuracy, as exemplified by the Veillonella-related dysbiosis and Phascolarctobacterium associations in depression. Future research should explore whether the bacterial taxa we identified in the gut are also reflected in oral microbiome compositions and track longitudinal changes in relation to depression progression and treatment response.

The most significant and innovative aspect of our study is the predicted dichotomy in metabolic pathways. The deceased group was dominated by pathways associated with infectious processes, inflammation, and antibiotic resistance, while the control group exhibited the prevalence of energy metabolism and vitamin synthesis pathways.

Our principal component analysis clearly demonstrates these relationships between gut microbiome composition, metabolic pathways, and depression indicators. Subjects with higher depression scores correlate with increased content of *Dorea formicigenerans* and *Firmicutes*. In contrast, healthy metabolic profiles, characterized by energy metabolism and metabolism of cofactors and vitamins, are positioned in the opposite direction, confirming the negative correlation with depression severity observed in our other analyses.

Particularly important is the identification of predicted-reduced activity of the Krebs cycle (c = −0.4, *p* < 0.001) and overall energy metabolism (c = −0.5, *p* = 0.006) in depression and suicidal behavior. These findings are consistent with the mitochondrial hypothesis of depression (Allen et al., 2018) [24], but our study is the first to link these metabolic changes to specific taxonomic shifts in the microbiome. For instance, the strongest negative correlations were observed between the family *Lachnospiraceae* and carbon fixation pathways (−0.8), as well as between the order *Lachnospirales* and the Krebs cycle (−0.8), suggesting a direct link between taxonomic changes and metabolic disturbances.

The increased representation of the pentose phosphate pathway (*p* < 0.001) in the deceased group is of particular interest from a pathophysiological perspective. This pathway is the primary source of NADPH necessary for protection against oxidative stress, and its activation may represent a compensatory mechanism in response to increased oxidative stress in depression and suicidal behavior. On the other hand, its activation may also reflect increased energy demands under conditions of inflammation and oxidative stress characteristic of severe depressive states. Głombik et al. (2020) [25] point to disruptions in energy metabolism, including the pentose phosphate pathway, in depression, suggesting that its activation may reflect not only adaptive processes but also imbalances in neuronal energy metabolism regulation, which may contribute to the pathophysiology of depressive and suicidal states.

Our study expands the understanding of the “gut-brain axis” in the context of mental disorders. Traditionally, the influence of the microbiome on the brain was considered to be mediated predominantly through the vagus nerve, immune system, and neurotransmitters (Cryan et al., 2019) [26]. Our results, however, point to the potential role of metabolic disturbances, especially in energy metabolism, as another key mechanism of this interaction.

The negative correlation we found between depression and vitamin B6 metabolism (c = −0.2, *p* = 0.025) is consistent with data on B vitamin deficiency in depression (Skarupski et al., 2010) [27], but our study is the first to suggest the role of the microbiome in this association. Vitamin B6 is a cofactor for the synthesis of several neurotransmitters, including serotonin, dopamine, and GABA, and its deficiency may contribute to the development of depression.

Analyzing the taxonomic composition of the microbiome in the context of predicted metabolic activity, it is important to note that representatives of the phylum Bacteroidetes, which were reduced in groups with depression and suicidal behavior, are the main producers of B vitamins in the intestine, while representatives of Firmicutes act predominantly as consumers of these vitamins [28]. According to recent estimates, up to 86% of the daily requirement of vitamin B6 can be covered by gut microbiota [29], which emphasizes the potentially significant role of microbiome changes in providing the body with this important cofactor.

One of the most significant results of our study is the establishment of specific microbiome profiles associated not only with depressive symptoms but also with suicidal behavior. While previous studies have focused primarily on the relationship between the microbiome and depression [1,13], our work is the first to comprehensively investigate microbiome markers of suicide risk.

Particularly important is the identification of taxa strongly associated with suicidal intentions according to the CSSRS scale, including *Streptococcus mutans*, *Streptococcus salivarius*, and *Lactobacillus rhamnosus*. Interestingly, *Streptococcus mutans* and *Streptococcus salivarius* are traditionally associated with the oral cavity rather than the intestine, which may indicate the role of oral microbiota in the pathogenesis of depression and suicidal behavior, an aspect rarely considered in previous studies.

Unlike the work of Maes et al. (2023), which suggested a possible association between the microbiome and suicidal behavior based on theoretical models, our study presents the first empirical data confirming this connection [10]. Furthermore, we identified specific bacterial taxa and metabolic pathways potentially involved in this association, opening new perspectives for understanding the biological foundations of suicidal behavior.

Despite the significance of the obtained results, our study has several limitations that should be considered when interpreting the data. The relatively small sample size (36 deceased cases and 35 controls) may limit the statistical power of the study, especially for the analysis of rare taxa. Additionally, the substantial difference in gender distribution between groups (men predominated in the deceased group, while women predominated in the control group) may influence the results, even after adjustment for this confounding factor, considering known gender differences in microbiome composition [30]. The cross-sectional nature of the study does not allow for the establishment of causal relationships between changes in the microbiome and depression/suicidal behavior. It remains unclear whether the observed microbiome changes are a cause or consequence of depressive states. Also, in the deceased group, samples were obtained posthumously, which may affect the composition and functional characteristics of the microbiome due to postmortem changes in the intestinal environment [31]. Although samples were collected immediately after bodies were received and we accounted for the time between death and sample collection in our statistical analysis, the time interval between death and sample collection varies, potentially affecting the results. Additionally, the study did not systematically assess glycemic status or signs of diabetes/prediabetes during autopsies, which could have provided valuable insights into metabolic abnormalities potentially associated with the observed microbiome changes, particularly the increased pentose phosphate pathway activity and Streptococcus prevalence. The study could not account for dietary habits, physical activity, and other lifestyle factors for the suicide group, which can significantly influence microbiome composition [32]. These factors may be particularly important considering the known effects of depression on appetite and eating behavior. Additionally, the 16S rDNA analysis used in the study has limitations in taxonomic resolution and does not allow for the identification of bacteria at the strain level, which may have different functional properties even within the same species. Furthermore, prediction of metabolic pathways based on 16S data is indirect and may not accurately reflect the actual metabolic activity of the microbiome. It is important to note significant differences between groups in the frequency of alcohol consumption and smoking, which can be both risk factors for suicidal behavior and factors influencing microbiome composition. For instance, smoking is associated with changes in microbiota composition and may exacerbate dysbiosis, while alcohol consumption is linked to increased intestinal barrier permeability and inflammatory changes. Future studies need to more thoroughly examine the contribution of these factors to the formation of specific microbiome profiles in depression and suicidal behavior.

Despite these limitations, our study provides valuable data on the relationship between the gut microbiome, depression, and suicidal behavior, opening new perspectives for understanding the biological foundations of these conditions and developing innovative therapeutic approaches.

## 4. Materials and Methods

### 4.1. Recruitment and Screening of Study Subjects

This prospective study included 35 volunteers for the “Alive cntrl” group, which consisted of both individuals with varying degrees of depressive symptomatology (n = 17) and healthy participants without signs of depression (n = 18). The second group comprised 36 cases of completed suicide (“Deceased case”). Autopsy material was obtained by forensic medical examiners from the Branch of the Institute of Forensic Examinations of the RGKP “Center for Forensic Examinations of the Ministry of Justice of the Republic of Kazakhstan” in cases of suicide (hanging, stab wounds, etc.). The demographic and clinical characteristics of the study participants are presented in Table 1.

All materials were collected with official permission from the Institution’s administration, law enforcement agencies, and relatives of the deceased. A special Informed Consent form was developed for relatives regarding participation and collection of biological samples for scientific research purposes.

The study received approval from the Ethics Committee of the Center for Life Sciences at “National Laboratory Astana” (Protocol No. 05-2022 dated 21 October 2022). All data were used only with patients’ consent. Personal identification data were completely anonymized.

Screening and recruitment of the control group and the group of patients with depressive disorders were conducted at the clinics of “University Medical Center” of Nazarbayev University and the Neurology Department of “Multidisciplinary City Hospital No. 1” in Astana. The following validated instruments were used to assess depressive states and suicidal intentions: Beck Depression Inventory (BDI); Hamilton Rating Scale for Depression (HDRS; Zung Self-Rating Depression Scale (ZDRS); Hospital Anxiety and Depression Scale (HADS) with subscales for anxiety (HADS-A) and depression (HADS-D); Clinical Questionnaire for Identifying and Evaluating Neurotic States (K.K. Yakhin-D.M. Mendelevich) with the following subscales: Anxiety Scale (YMA); Neurotic Depression Scale (YMND); Asthenia Scale (YMNAs); Hysterical Response Type Scale (YMHR); Obsessive–Phobic Disorders Scale (YMOPD); Autonomic Disorders Scale (YMAD); Summary Score (YMSUM); Columbia-Suicide Severity Rating Scale (C-SSRS).

Inclusion criteria for the “Alive cntrl” group were age 18–78 years, ability to provide informed consent, and absence of severe somatic diseases. This group included both participants with signs of depression of varying severity and healthy volunteers without depressive symptoms to form a continuum of depressive symptom severity. Exclusion criteria were presence of psychotic disorders, bipolar disorder, severe neurological diseases, inflammatory bowel diseases, and antibiotic use during the preceding 3 months.

### 4.2. Fecal Sample Collection and DNA Extraction

Fecal samples were collected in tubes containing DNA/RNA stabilizing solution (DNA/RNA Shield Collection Tube, Zymo Research, R1101, Irvine, CA, USA) and stored and transported at +4 °C until DNA isolation. Fecal samples in the “Deceased case” group were collected during forensic medical examination under sterile conditions from the lower third of the sigmoid colon. To minimize contamination, samples were collected before abdominal cavity dissection of other organs. Fecal samples were immediately placed in tubes containing DNA/RNA stabilizing solution.

For each sample, approximately 1 g of fecal material was collected using the provided collection spoon and placed into tubes containing 10 mL of DNA/RNA Shield solution. Tubes were tightly capped and thoroughly mixed by inverting 10 times to create a homogeneous suspension. This procedure ensured proper sample preservation and stabilization of nucleic acids. For the “Deceased case” group, the same amount of sample was collected from the lower third of the sigmoid colon following the identical protocol.

To minimize the effect of postmortem changes on microbiome composition, samples in the “Deceased case” group were collected within the first 24 h after death confirmation (mean time from death confirmation to sample collection was 16.1 ± 7.8 h, Supplementary File S1). Time from death to sample collection was considered as a covariate in statistical analysis.

Total genomic DNA was extracted using the ZymoBIOMICS DNA Miniprep Kit (Zymo Research, D4300, Irvine, CA, USA), with sterile µQ water included as a negative extraction control. ZymoBIOMICS Microbial Community Standard (Zymo Research, D6300) was used as a positive control to validate the extraction method, PCR amplification, and sequencing procedures. DNA quality was evaluated by measuring the OD260/280 ratio with a Nanodrop spectrophotometer and by visualization on 1.0% agarose gel electrophoresis. DNA concentration and purity were quantified using a Qubit 3.0 fluorometer (Thermo Fisher Scientific, Waltham, MA, USA). In addition, 16S Amplicon Metagenomic Sequencing was conducted both internally using the Illumina NovaSeq6000 platform following metagenomic sequencing protocols, and externally at Novogene laboratory (Beijing, China) also utilizing the Illumina NovaSeq 6000 platform according to the manufacturer’s standard procedures. Initial bioinformatic analysis was completed using LotuS2 (less operational taxonomic unit scripts 2).

### 4.3. Statistical Analysis and Visualization

Statistical analysis and visualization were performed in Python v3.12 using NumPy v2.0.1, SciPy v1.15.1, statsmodels v0.14.4, scikit-learn v1.6.1, Matplotlib v3.10.0, and seaborn v0.13.2 packages. Biodiversity analysis was performed using scikit-bio v0.6.3. The depth of taxonomic tables did not differ significantly between the alive and deceased groups (171,113 ± 9517 vs. 171,613 ± 9878 assigner reads, respectively, *p* = 0.82, Ind. *t*-test). Taxonomic data were rarified prior to analysis. The Adonis2 package for R v.4.4.2 was used to estimate the significance of grouping for beta analysis. Baseline characteristics of the groups were compared using the one-sample *t*-test, Mann–Whitney U rank test, and Fisher’s exact tests where appropriate. Association analysis between bacterial taxa/pathways and psychometric tests was performed using generalized linear regression (GLM), adjusting for age and sex. Differential analysis (difference between the alive and deceased groups) was performed using GLM, adjusting for age, sex, and time from death to sample collection (in hours). In GLM analysis, markers were considered significant with *p* < 0.05 and Cliff’s d effect > 0.2. For living participants, time passed from death to sample collection was set to 0. Only features with a prevalence of 25% and 50% (both groups) were considered for association and differential analysis, respectively. Between-group diversity was assessed using weighted Bray–Curtis distances. Adonis2 with 999 permutations was used to assess the significance of grouping and parameter effect using marginal regression. Within-sample diversity was assessed using the Shannon and Faith indices. KEGG pathways were aggregated to higher levels using total sum scaling (TSS). Correlation analysis between marker taxa and pathway was performed using Spearman’s coefficient. Depression scores were aggregated into a single parameter using principal component analysis (PCA) on standardized data. The aggregated parameter (PCA # 1) accounted for 57% of the variation and was used to represent the “depression scale” in differential analysis.

## 5. Conclusions

The study results demonstrate a distinct relationship between gut microbiome composition, depressive states, and suicide cases. It is important to note that all identified taxa significantly elevated in the deceased group also demonstrated positive correlations with the severity of depressive symptoms in the control group. This indicates a continuous spectrum of associations between microbiome changes, depression, and suicide risk.

The deceased group is dominated by metabolic pathways associated with infectious processes, inflammation, and antibiotic resistance, while the control group shows the prevalence of energy metabolism and vitamin synthesis pathways. This pattern of functional changes may reflect potential mechanisms of the relationship between gut dysbiosis and the pathophysiology of depression and suicidal behavior.

The study supports the hypothesis regarding potential mechanisms of interaction between gut dysbiosis, disruptions in energy metabolism, and inflammatory processes in depression and suicidal behavior, providing new data for understanding the “gut-brain axis” in the context of psychiatric disorders.

## Figures and Tables

**Table 1:** Demographic, Clinical, and Psychometric Characteristics of the Study Groups.

	Alive Cntrl	Deceased Case
**Demographic characteristics**		
N	35	36
Age (yrs), M ± Sd	36.5 ± 14.7	42.7 ± 18.0
Sex (f/m), n	24/11	7/29
Smoking, n	2/35	22/36
Alcohol, n	3/35	16/36
Drugs, n	0/35	3/36
**Distribution by severity of psychometric indicators**		
**Beck Depression Inventory (BDI)**		Psychometric measures for the “Deceased case” group could not be collected due to the nature of this group (data obtained posthumously). Information regarding the psychiatric status of these cases was obtained from medical records and relatives′ reports; however, standardized psychometric testing was not conducted.
Normal	29	
Mild depression	3	
Moderate depression	2	
Severe depression	1	
**Hamilton Depression Rating Scale (HDRS)**		
Normal	17	
Mild depression	11	
Moderate depression	6	
Severe depression	0	
**Zung Self-Rating Depression Scale (ZDRS)**		
Normal	25	
Mild depression	10	
Moderate depression	0	
Severe depression	0	
**Hospital Anxiety and Depression Scale**		
**HADSA (anxiety)**		
Normal	24	
Subclinical anxiety	3	
Clinical anxiety	8	
**HADSD (depression)**		
Normal	27	
Subclinical depression	5	
Clinical depression	3	
**Yakhin–Mendelevich Clinical Questionnaire**		
YMA (anxiety scale)		
Normal	20	
Subnormal	15	
**YMND (neurotic depression scale)**		
Normal	16	
Subnormal	18	
YMNAs (asthenia scale)		
Normal	21	
Subnormal	14	
**YMHR (hysterical response type scale)**		
Normal	17	
Subnormal	18	
**YMOPD (obsessive–phobic disorders scale)**		
Normal	19	
Subnormal	16	
**YMAD (autonomic disorders scale)**		
Normal	23	
Subnormal	12	
YMSUM (total score)		
Normal	19	
Subnormal	16	
**Columbia Suicide Severity Rating Scale (CSSRS)**		
No	32	
Yes	3	

## Data Availability

Raw sequencing data and associated sample metadata will be provided upon reasonable request.

## Supplementary Materials

The following supporting information can be downloaded at: https://www.mdpi.com/article/10.3390/ijms26104880/s1.
